# Structured multicellular intestinal spheroids (SMIS) as a standardized model for infection biology

**DOI:** 10.1186/s13099-024-00644-6

**Published:** 2024-09-17

**Authors:** Angelina Kraski, Paweł Migdał, Robert Klopfleisch, Clara Räckel, Jutta Sharbati, Markus M. Heimesaat, Thomas Alter, Carlos Hanisch, Greta Gölz, Ralf Einspanier, Soroush Sharbati

**Affiliations:** 1 Institute of Veterinary Biochemistry, Freie Universität Berlin, Berlin, Germany; 2 Institute of Animal Husbandry and Breeding, Wrocław University of Environmental and Life Sciences, Wrocław, Poland; 3 Institute of Veterinary Pathology, Freie Universität Berlin, Berlin, Germany; 4 School of Science, OSZ Lise Meitner, Berlin, Germany; 5 Institute of Microbiology, Infectious Diseases and Immunology, Charité, Universitätsmedizin Berlin, Corporate Member of Freie Universität Berlin, Humboldt-Universität Zu Berlin, and Berlin Institute of Health, Berlin, Germany; 6 Institute of Food Safety and Food Hygiene, Freie Universität Berlin, Berlin, Germany; 7 SGS INSTITUT FRESENIUS GmbH, Berlin, Germany

**Keywords:** Multilayered spheroid, 3D cell culture, Intestinal mucosa, Infection model

## Abstract

**Background:**

3D cell culture models have recently garnered increasing attention for replicating organ microarchitecture and eliciting in vivo-like responses, holding significant promise across various biological disciplines. Broadly, 3D cell culture encompasses organoids as well as single- and multicellular spheroids. While the latter have found successful applications in tumor research, there is a notable scarcity of standardized intestinal models for infection biology that mimic the microarchitecture of the intestine. Hence, this study aimed to develop structured multicellular intestinal spheroids (SMIS) specifically tailored for studying molecular basis of infection by intestinal pathogens.

**Results:**

We have successfully engineered human SMIS comprising four relevant cell types, featuring a fibroblast core enveloped by an outer monolayer of enterocytes and goblet cells along with monocytic cells. These SMIS effectively emulate the in vivo architecture of the intestinal mucosal surface and manifest differentiated morphological characteristics, including the presence of microvilli, within a mere two days of culture. Through analysis of various differentiation factors, we have illustrated that these spheroids attain heightened levels of differentiation compared to 2D monolayers. Moreover, SMIS serve as an optimized intestinal infection model, surpassing the capabilities of traditional 2D cultures, and exhibit a regulatory pattern of immunological markers similar to in vivo infections after *Campylobacter jejuni* infection. Notably, our protocol extends beyond human spheroids, demonstrating adaptability to other species such as mice and pigs.

**Conclusion:**

Based on the rapid attainment of enhanced differentiation states, coupled with the emergence of functional brush border features, increased cellular complexity, and replication of the intestinal mucosal microarchitecture, which allows for exposure studies via the medium, we are confident that our innovative SMIS model surpasses conventional cell culture methods as a superior model. Moreover, it offers advantages over stem cell-derived organoids due to scalability and standardization capabilities of the protocol. By showcasing differentiated morphological attributes, our model provides an optimal platform for diverse applications. Furthermore, the investigated differences of several immunological factors compared to monotypic monolayers after *Campylobacter jejuni* infection underline the refinement of our spheroid model, which closely mimics important features of in vivo infections.

**Supplementary Information:**

The online version contains supplementary material available at 10.1186/s13099-024-00644-6.

## Background

For many years, cell culture models have been a valuable tool for understanding complex molecular and cellular mechanisms in both basic and applied research. Using appropriate in vitro models, new and in-depth insights into cell biology for normal differentiation and pathogenesis have been gained [1, 2]. They also play a crucial role in investigating the pharmacokinetics and pharmacodynamics of drugs, conducting toxicity testing for various substances, and contributing to the emerging field of tissue engineering and regenerative medicine [3, 4]. The majority of in vitro experiments mentioned were originally performed as two-dimensional (2D) single cell or co-culture models, as they are inexpensive, easy to handle, reproducible and efficient [3, 5, 6]. Such models have clear limitations, as they can only reproduce complex tissue structures to a very limited extent and therefore often do not reflect the complexity of human or animal tissue physiology [6]. Especially concerning cell–cell interactions and extracellular microenvironments, 2D cell monolayers are inadequate for accurate representation [1]. While animal models offer a more comprehensive understanding of human physiology and pathophysiology they come with drawbacks. These include time-consuming nature of experiments, high costs, and limitations arising from species-specific differences. Additionally, significant inter-individual variability and ethical concerns regarding the welfare of animals utilized cannot be overlooked [7, 8].

Hence, three-dimensional (3D) cell culture models have gained increasing importance in recent decades. In these models, cells are cultured under conditions that allow them to grow and interact in all three spatial dimensions, closely resembling in vivo microstructures. As a result, 3D cell culture models bridge the gap between traditional 2D cultures and in vivo models, offering a promising path for overcoming the challenges previously mentioned. Moreover, these optimized models facilitate the development of cell–cell and cell-extracellular matrix interactions, resulting in tissue architectures that closely resemble the in vivo situation [9]. In line with the 3R principle (reduction, replacement and refinement of animal experiments), they not only reduce the number of animal experiments but also adequately reproduce in vivo data by more closely mimicking organ morphology and physiology.

3D cell culture can mainly be subdivided into organoids and spheroids. Organoids are derived either from (induced) pluripotent stem cells or from organ-specific adult stem cells obtained after tissue biopsies. As soon as they differentiate into tissue-specific and specialized cell types in culture, they organize themselves independently into organ-like structures [10, 11]. This includes, for example, the formation of typical tissue-specific protrusions such as crypts or villi in intestinal organoids [12, 13]. However, these mini-organs obtained from patients or individual donors have some disadvantages. They require human or animal biopsies, are relatively expensive to produce, time-consuming and the harmonization and standardization of the method is limited [14]. In this context, the accessibility of biopsies and the presence of inter-individual differences pose particular challenges, as organoids are derived from individual donors. On the other hand, this has advantages, as greater genetic diversity enables reliable and realistic results. The orientation of the mini-organ, which is usually embedded in a matrix, plays an important role. This applies in particular to the investigation of molecular aspects of infections. For instance, in intestinal organoids, the luminal surface is typically oriented towards the interior, posing challenges for exposing it to luminal factors like microbiota and pathogens. The reversal of orientation has rarely been performed, takes a long time and is a complex and labor-intensive procedure. Therefore, their use as standardized infection models or for high-throughput procedures is limited [15]. However, studies have been conducted utilizing apical-out orientated organoids as an advanced model, applied in infection research [15–18]. Spheroids, on the other hand, are formed from cell cultures or biopsy material in classic hanging drops or by using non-adhesive surfaces leading to aggregation of cells and the formation of 3D cell clusters [19–21]. Spheroids are spherical, self-organizing structures that can either be embedded in an extracellular matrix (e.g., Matrigel) or maintained without a matrix, floating freely in culture [22]. Spheroids facilitate 3D cell–cell interactions, closely resembling in vivo environments. They are cost-effective and straightforward to generate, offering reproducibility and ease of standardization and harmonization. They are distinguished in homotypic and multicellular (heterotypic) spheroid models. Homotypic spheroids consist of only one cell type, multicellular models of two or more cell types. However, the multicellular spheroid models have so far lacked a structured cellular composition and microarchitecture. They have been successfully used for many years for cell biology studies, preferably in preclinical oncology research [23–25]. While lung spheroids have been used in SARS-CoV-2 research, they have not yet been extensively studied as standardized bacterial infection models [26, 27]. Bacterial infections pose significant challenges to global health, yet suitable 3D models capable of replacing animal models and analyzing the molecular mechanisms underlying host cell invasion and dissemination remain limited. Particularly concerning the gastrointestinal tract, an ideal model would need to feature a surface composed of differentiated enterocytes to be both functional and physiologically relevant while mimicking the 3D microarchitecture.

The mammalian intestinal epithelium is a rapidly self-renewing tissue. In homeostasis, Lgr5^+^ stem cells located at the base of the crypts, produce precursor cells of secretory and enterocytes that terminally differentiate into goblet cells, enteroendocrine cells or nutrient-absorbing enterocytes migrating upwards [28]. Notably, during tissue repair after injury, enterocytes within the intestinal crypts can dedifferentiate to an undifferentiated state, replacing the Lgr5^+^ stem cells and generating proliferative stem cells and Paneth-like secretory cells [28]. Various markers can be utilized, exhibiting altered expressions depending on the differentiation status of enterocytes along the crypt axis. The epidermal growth factor (EGF), a growth factor playing a crucial role in regulating cell proliferation and differentiation, is expressed in the microenvironment at the crypt basis [29, 30]. Further, ephrin receptors are predominantly expressed near the bottom of the crypts while Ephrin B1 and Ephrin B2 segregate differentiated from precursor cells in the villus and are dominantly prevalent there [29, 31, 32]. Ephrin receptors are the largest family of tyrosine kinase receptor. After ephrin binding, they facilitate contact-dependent communication between cells, whether of the same or different type, to regulate various cellular processes such as morphology, adhesion, movement, proliferation, survival, and differentiation [33]. Markers for fully differentiated and functional enterocytes are the transporters solute carrier family 5 member 1 (SLC5A1 or SGLT1) and peptide transporter 1 (SLC15A1 or PEPT1) [34]. While the sodium-dependent SGLT1 is primarily responsible for the absorption of dietary glucose and galactose from the intestinal lumen, PEPT1 facilitates the uptake of dipeptides and tripeptides into enterocytes. Both of these transporters are located in the brush border membrane of functional intestinal enterocytes [35, 36]. Ideally, intestinal 3D cell culture models should exhibit abundant expression of these markers to demonstrate their differentiation status and, consequently, their resemblance to the in vivo environment.

Here we have tackled the task of developing a suitable intestinal infection model that mimics the intestinal mucosal microarchitecture. In contrast to most previously reported intestinal spheroids, which are limited to only one type of cells [37, 38], the spheroids described here consist of up to four different cell types that self-assemble in an ordered fashion in a liquid medium. By integrating fibroblasts, enterocytes, goblet cells and monocytic cells, relevant players in bacterial infections were considered. In the loose connective tissue of the mucosa, fibroblasts are the predominant cell type, while enterocytes form the inner lining of the intestine. Goblet cells, responsible for producing protective mucus, further highlight the relevance of SMIS for studying intestinal infections. Monocytes are chosen as exemplary intestinal immune cells. The interactions between epithelial and immune cells constitute a crucial aspect of the immune response to infection, which are not replicated in conventional single-cell spheroids [39, 40]. We characterized the formed spheroids and compared them with conventional 2D cell cultures and an in vivo model in an infection-dependent context. In this study, spheroids were infected with *Campylobacter jejuni*, a significant zoonotic bacterium utilized in our previous research and selected as an exemplary pathogen. Various infection markers were tested and compared to those of 2D monolayers and in vivo infections. To demonstrate the versatility of the developed protocol, we have extended SMIS to the species mouse (*Mus musculus*) and pig (*Sus scrofa*).

## Results

### Assembly and characterization of human structured multicellular intestinal spheroids (SMIS)

The objective of this study was to design and characterize novel structured multicellular spheroids resembling the mammalian luminal intestinal surface. Given that most available spheroid models are either homotypic or lack specific structure, we initially experimented with creating a basic version of human SMIS using only primary fibroblasts (NHDF neo) and absorptive enterocytes (Caco-2). In mimicking the intestinal mucosa, fibroblasts first formed a spheric mesenchymal core, which was subsequently colonized with epithelial cells as a monocellular layer and incubated for two days. After only 24 h of culture, the core remained partially uncovered whereas slight structural instability was observed in the spheroids after five days in culture. This led to the decision to focus on the two-day colonization time for further analysis. After successfully generating the basic version, we further enhanced the model by incorporating a colonic goblet cell model (HT-29-MTX-E12) into the outer cell layer using the same protocol. This resulted in the formation of multicellular spheroids.

**Fig. 1:**
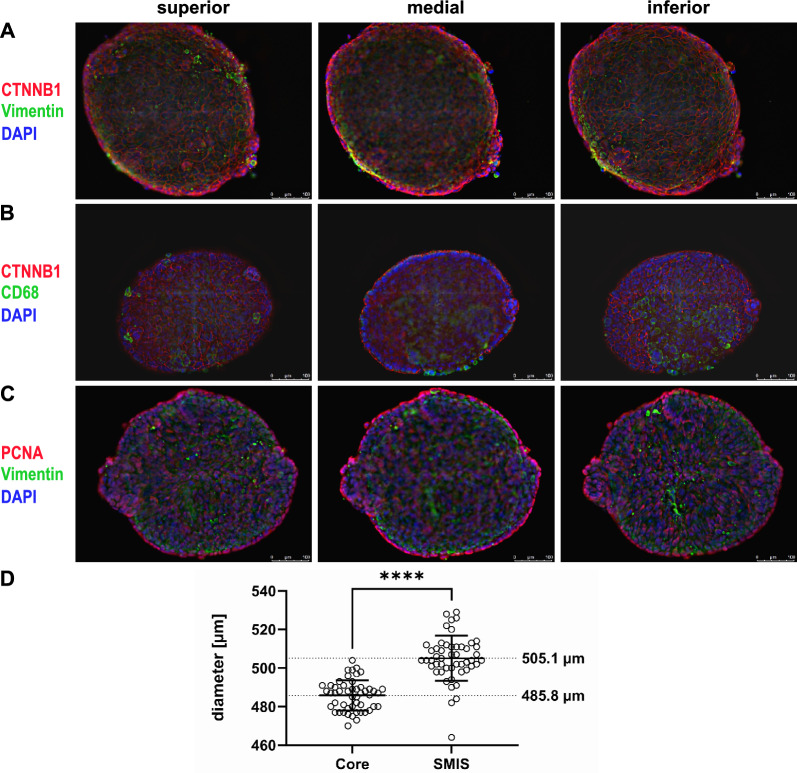
Structural architecture of human SMIS detected by Immunofluorescence staining and Z-stack projections. **A** Representative images of the localization of vimentin (green), adherens junctions between epithelial and goblet cells (CTNNB1, red) and nuclei (blue). **B** Staining of human SMIS after the implementation immune cells (THP-1) to the model. CD68 is stained green, CTNNB1 red and nuclei blue. **C** To identify active proliferation in the human spheroids, the proliferation marker PCNA was stained red. For a better overview, vimentin was stained green and nuclei blue. For green immunostaining DyLight 488 and for red staining DyLight 594 was used, whereas nuclei were stained blue by DAPI. Scale bars indicate 100 µm (magnification). Exposure time was identical for all spheroids and presented images are representative for 10 biological replicates tested. **D** Diameter distribution of 50 human fibroblast cores and 50 human SMIS. The charts display each individual measurement with mean ± SD (bars)

To characterize and ensure accurate formation of SMIS, immunofluorescent staining (IF) was conducted. Each staining procedure was replicated, with a minimum of 10 spheroids examined in each instance, yielding consistent results to ensure reproducibility. Here, fibroblasts were stained using vimentin, a mesenchymal marker expressed in the fibroblasts core, while adherens junctions between intestinal epithelial and goblet cells were identified by CTNNB1 (β-Catenin) immunostaining. Z-stack microscopy yielded images of different focal layers that revealed significant findings. Predominantly, a monolayer of isoprismatic enterocytes and goblet cells was observed, arranged around and covering the entire spheric fibroblast core. This configuration facilitated direct contact between the epithelial cell layer and the underlying fibroblasts (Fig. 1A). The successful generation of structured multicellular spheroids facilitated interactions between enterocytes and mesenchymal cells as well as between enterocytes and goblet cells. However, some green-positive cells were visible at the edge of the SMIS. For additional observations, single staining images for each channel are provided in Additional Fig. 1. Finally, we expanded the model to include four relevant intestinal cell types by integrating the monocytic cell line A-THP-1. Immunostaining with Cluster of Differentiation 68 (CD68), a surface protein expressed in monocytes and macrophages, allowed us to visualize the localization of the immune cells (Fig. 1B). The surface areas of the spheroids were completely lined with epithelial cells, goblet cells and monocytes. This arrangement facilitated the development of immune-enterocyte and immune-mesenchymal interactions, in addition to those mentioned earlier. To distinguish between proliferating and non-proliferating cells, SMIS were stained with the proliferation marker Proliferating-Cell-Nuclear-Antigen (PCNA). As depicted, the majority of proliferating cells was detected on the external side facing the environment (Fig. 1C). No proliferation was detected in the fibroblast core.

To evaluate the scalability and of the spheroids as well as standardization of the protocol, we measured the diameter of 50 fibroblast cores and subsequently 50 multicellular spheroids. The fibroblast cores exhibited an average diameter of 485.8 µm (± 7.8 µm), while the multicellular spheroids, featuring an epithelial monolayer, measured 505.1 µm (± 11.7 µm). Data sets of the diameter distribution including standard deviation within the measured population can be found in Fig. 1D. Standardization of SMIS was ensured by utilizing cells from similar passages across all experiments. Our capacity allowed for the formation of up to 180 spheroids simultaneously, with the limiting factor being the availability of cells rather than the spheroid formation process.

### Characterization of SMIS by electron microscopy

**Fig. 2:**
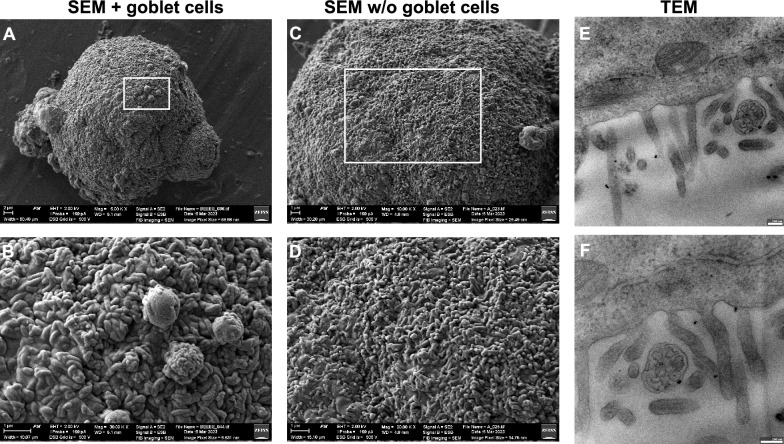
Scanning and transmission electron microscopy of human SMIS. **A** SEMs display the surface of the spheroids consisting of a fibroblast core, covered by epithelial and goblet cells. Scale bars indicate 2 µM. **B** Higher magnification of the SEMs, scale bars indicate 1 µM. **C** Analysis of the surface of human SMIS with only fibroblasts and epithelial cells by SEM. Scale bars indicate 1 µM. **D** SEMs in higher magnification, scale bars indicate 1 µM. **E** Transmission electron microscopy of the spheroids consisting of fibroblasts, epithelial and goblet cells to identify membrane protrusions proving microvilli formation. Scale bars indicate 200 nm. **F** Higher magnification of the TEMs, scale bars indicate 200 nm

Next, we conducted a more detailed examination of the surface of the spheroids and their degree of differentiation, exemplified by scanning electron microscopy (SEM) and transmission electron microscopy (TEM). As shown in Fig. 2, the surface of SMIS including goblet cells (Fig. 2A, B) and without goblet cells (Fig. 2C, D) was covered by small, outgrowth structures, potentially identified as microvilli. Microvilli are miniature, uniform, finger-like protrusions of the cell membrane in enterocytes, facing the intestinal lumen. They are regularly ordered to form brush borders, which serve to enhance absorption [41]. In the SEMs of our human model, we observed finger-like protrusions, although they were irregularly arranged and varied in length. These SEM observations suggested that the spheroids exhibited dysplastic (not fully developed) microvilli produced by the Caco-2 cells. Individual cellular membrane protrusions were visible in the TEM images of the SMIS with goblet cells, confirming our observation regarding microvilli and verifying the nature of the finger-like structures as microvilli (Fig. 2E, F). Furthermore, the observed lengths of the microvilli presented here are consistent with the lengths found in vivo, typically ranging from 1 to 3 µm [41]. While the microvilli may not appear densely packed and aligned as they do in vivo, SMIS seem to form a brush border to some extent within 2 days after assembly.

### Intestinal epithelial layer in SMIS shows significantly earlier differentiation than conventional 2D cell cultures

**Fig. 3:**
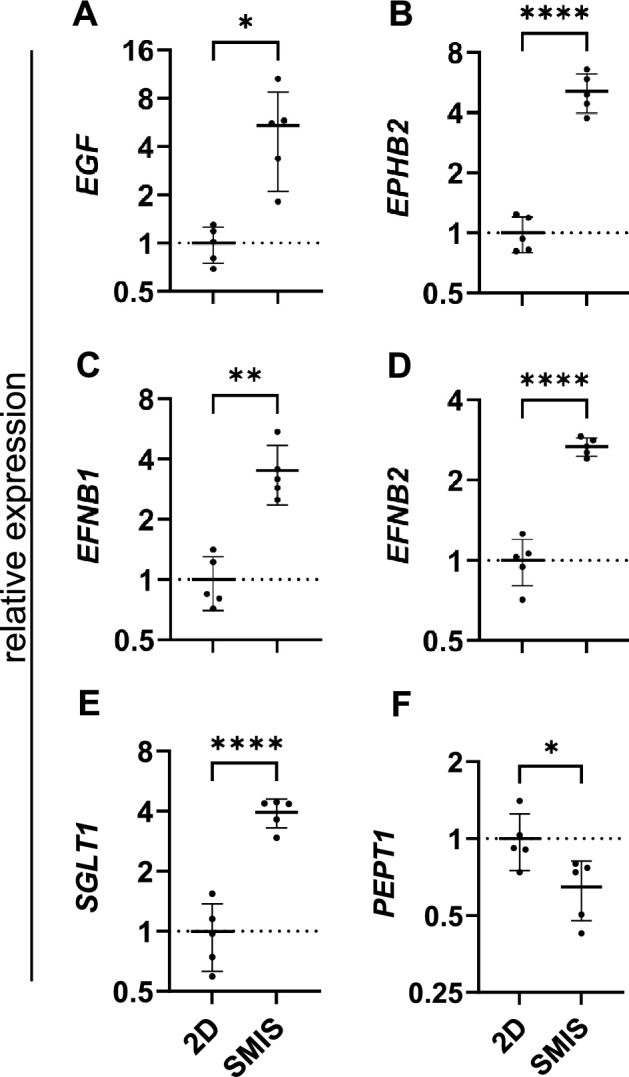
Relative gene expression of relevant differentiation and proliferation factors of human SMIS compared to Caco-2 monolayers. **A** Expression of the proliferation factor *EGF* is upregulated in the spheroids. **B** mRNA levels of the proliferation marker *EPHB2* are increased in SMIS. **C** Gene expression analysis revealed elevated levels of the differentiation marker *EFNB1* in the 3D model. **D** Intracellular *EFNB2* is upregulated in the spheroids. **E** Absorptive capacities of the SMIS were assessed by increased *SGLT1* expression. **F** As the only downregulated factor, *PEPT1* levels were decreased in the 3D intestinal model. All expressions were relatively calculated to 2D Caco-2 monolayers and normalized with HPRT, PPIB and B2M. Charts show mean ± SD (bars) of each sample in triplicate measurements with five biological replicates. For each SMIS sample, 15 spheroids were pooled. Statistical significance is presented by asterisks compared to 2D monolayers. *P ≤ 0.05, **P ≤ 0.01, ****P ≤ 0.0001, unpaired t-test

The intestinal epithelium of mammals forms a constantly renewing system with proliferating cells at the bottom of the crypts, migrating upward, differentiating into absorbing enterocytes with microvilli that form a brush border [41, 42]. After electron microscopy revealed the formation of microvilli on the surface of the spheroids, we hypothesized that the epithelial cells incorporated into SMIS show earlier differentiation compared to Caco-2 monolayers cultured under the same conditions for two days. To validate this assumption and assess the physiological relevance of the spheroids, we examined the expression of several markers associated with proliferating and fully differentiated epithelial cells in both SMIS and conventional 2D Caco-2 cultures. Epidermal growth factor (EGF) and ephrin B receptor 2 (EPHB2) were chosen as markers for proliferating enterocytes [43–45]. In the 3D model, both markers exhibited a significant approximately five-fold increase in expression compared to the Caco-2 monolayer (Fig. 3A, B). EphrinB1 (EFNB1) and EphrinB2 (EFNB2) were also examined as markers present in differentiated enterocytes. Similarly, mRNA levels of both genes exhibited a significant increase in the spheroids, with *EFNB1* increasing by 3.5-fold (P ≤ 0.01) and *EFNB2* by 2.7-fold (P ≤ 0.0001) (Fig. 3C, D). Additionally, to assess the absorptive capacities of Caco-2 cells in both 3D and 2D cultures, we analyzed the expression of sodium-dependent glucose cotransporter 1 (SGLT1) and peptide transporter 1 (PEPT1), solute carrier proteins localized in the brush border of differentiated enterocytes. Our analyses revealed that *SGLT1* was significantly upregulated by 3.9-fold in the spheroids (P ≤ 0.0001), while surprisingly, *PEPT1* was significantly reduced by 0.64-fold (P ≤ 0.05) compared to the 2D culture (Fig. 3E, F).

### Application of human SMIS for infection biology compared to 2D monolayers

**Fig. 4:**
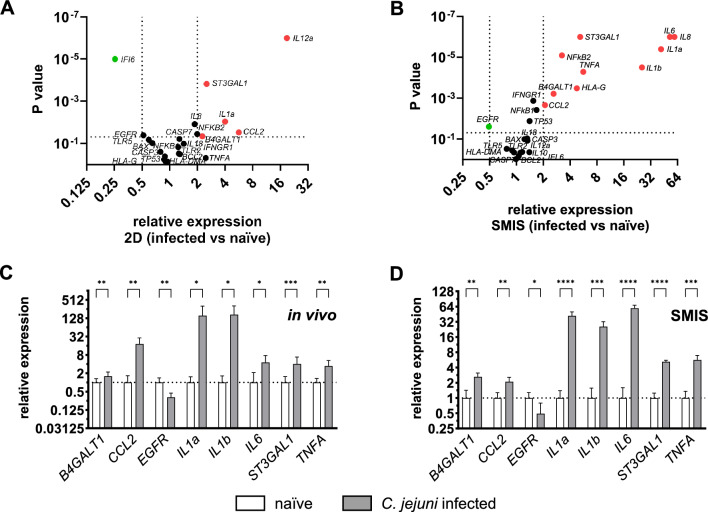
Gene expression analysis of immunological markers in human SMIS compared to single Caco-2 monolayers and to murine in vivo samples. **A** Volcano Plot of differently expressed immunological genes in *C. jejuni* infected compared to naïve Caco-2 cells. Only six factors are significantly regulated in infected monolayers, highest upregulation can be detected in *IL12a* whereas downregulation in *IFI6*. Expressions were relatively calculated to naive 2D controls. **B** Gene expression analysis of the immunological factors in *C. jejuni* infected SMIS, relatively calculated to naïve SMIS and presented in a Volcano Plot. Intracellular mRNA levels of eleven factors are significantly regulated in the infected samples. Most dominantly upregulation can be detected in the interleukins *IL8*, *IL6*, *IL1b* and *IL1a* whereas only levels of *EGFR* are significantly decreased. The infection experiments were conducted once, consisting of five independent biological replicates. Expressions of all targets shown in the volcano plots are normalized by HPRT, PPIB and B2M. Marked in red are all significantly regulated factors that are upregulated by two or more and in green all factors that are downregulated by 0.5 or less. Dots in the plots show the mean of all samples (n = 5) in triplicate measurements for five biological replicates. For each 3D sample, 15 SMIS were pooled. For statistical significance, unpaired t-tests were performed. **C** Relative gene expression of the significantly regulated immunological factors murine *C. jejuni* infected colonic tissue samples. All factors that are significantly upregulated in 3D are also significantly increased in the in vivo samples whereas mRNA levels of *EGFR* are significantly decreased in both. **D** Gene expression analysis of significantly regulated immunological factors in infected 3D SMIS. Bars show mean + SD of all samples (n = 5 for the spheroids and n = 19 in the colonic tissue samples) in triplicate measurements. For each 3D sample, 15 SMIS were pooled. Statistical significance is presented by asterisks. *P ≤ 0.05, **P ≤ 0.01, ***P ≤ 0.001, ****P ≤ 0.0001, unpaired t-test

While there are several suitable 3D intestinal infection models available, they are labor-intensive and have limited scalability. In vivo animal models are still widely used but raise ethical concerns and are complex in nature. Following the 3R principles, we investigated whether SMIS could serve as a suitable infection model for intestinal campylobacteriosis, aiming to reduce and replace the use of animal models. Therefore, we have implemented infection assays as a functional assay in this study. Moreover, we compared our results with those obtained from conventional Caco-2 monolayers. To achieve this, we selected various immunological and infection-related markers based on our previous studies [46, 47]—categorized into subgroups: ‘Regulation of the Immune Response’ (NFKB1, NFKB2, HLA-G, HLA-DMA, EGFR, TLR2, TLR5), ‘Cytokine Response’ (CCL2, IL1a, IL1b, IL6, IL8, IL10, IL12a, IL18, IFI6, IFNGR1, TNFA), ‘Apoptosis’ (TP53, BAX, BCL2, CASP3, CASP7), and ‘*C. jejuni*-related’ (ST3GAL1, B4GALT1)—and analyzed them using gene expression analysis. The markers related to *C. jejuni* infections were identified and published in our previous studies [48, 49]. To determine underlying differences between the models, we first investigated these infection markers in the conventionally cultured Caco-2 monolayers (Fig. 4A). In infected Caco-2 monolayers, only six factors were significantly regulated possessing a foldchange below 0.5- or higher than twofold compared to their naïve counterpart. Most significantly regulated were *IL12a* (19-fold increase, P ≤ 0.0001), *IFI6* (0.25-fold decrease, P ≤ 0.0001) and *ST3GAL1* (2.5-fold increase P ≤ 0.001). We then compared the infection markers in SMIS infected with *C. jejuni* compared to naïve SMIS. Gene expression analysis revealed 11 significantly regulated targets possessing a foldchange below 0.5- or higher than twofold compared to non-infected controls (Fig. 4B). Interleukins *IL6* (59-fold, P ≤ 0.0001), *IL8* (53-fold, P ≤ 0.0001), *IL1*a (42-fold, P ≤ 0.0001) and *IL1b* (25-fold, P ≤ 0.0001) showed the most strikingly increased foldchanges. In addition, mRNA levels of *ST3GAL1* (fivefold, P ≤ 0.0001), *NFKB2* (threefold, P ≤ 0.0001) and *TNFA* (sixfold, P ≤ 0.0001) were strongly increased. Only *EGFR* was significantly downregulated by a factor of 0.5 (P ≤ 0.05). We also analyzed differences in the expression of the targets in SMIS compared to 2D under naïve conditions as well as after *C. jejuni* infection. Numerous genes from all categories exhibited differential regulation in the spheroids compared to their 2D counterparts (Additional Fig. 2). Immunological markers were more prominently expressed in SMIS than in 2D. Nevertheless, the regulation of *IL18* (0.45-fold, P ≤ 0.001) was significantly reduced in the spheroids.

Overall, the amplitude of regulations was notably more pronounced in SMIS for most factors than for the naïve counterparts. Additionally, a greater number of infection-relevant markers were regulated in 3D compared to 2D, highlighting the advantage of utilizing SMIS as suitable infection models over 2D monolayers.

### The infection data obtained from human SMIS closely resemble those observed in the murine in vivo model of human campylobacteriosis

To evaluate the potential of the human SMIS to reduce and replace animal studies as infection models, we compared the SMIS infection results to those obtained from *C. jejuni* infected murine colonic tissue samples. These murine samples were derived from a specific mouse model of human campylobacteriosis, as described in our previous study [49]. Naïve colonic tissue samples were utilized as controls. We focused on significantly regulated genes with a foldchange below 0.5 or higher than two from the previously mentioned infected versus naïve SMIS analysis. Consistent with the 3D results, *B4GALT1*, *CCL2*, *IL1a*, *IL1b*, *IL6*, *ST3GAL1* and *TNFA* were also significantly upregulated in the infected murine samples compared to their naïve counterparts except of *EGFR*, which was also downregulated (Fig. 4C). Highly upregulated expression was observed in both models in *IL1a* and *IL1b* (Fig. 4C, D). *IL8*, *NFKB2* and *HLA-G* were excluded from the analysis: *IL8* was below the detection limit in the in vivo samples, *NFKB2* showed no regulation, and *HLA-G* was not expressed in mice.

### The SMIS model can be adapted to the mouse and pig species

**Fig. 5:**
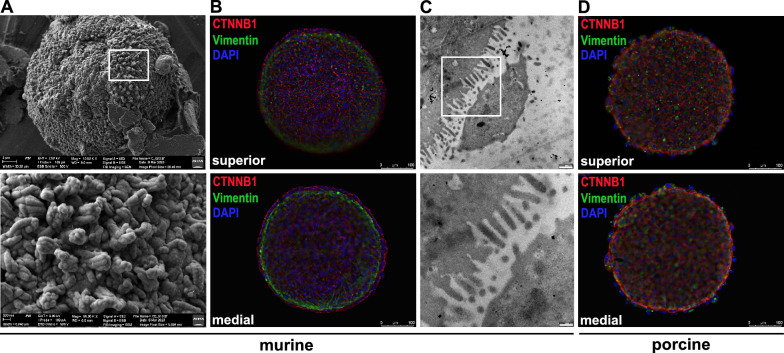
Characterization of murine and porcine SMIS. **A** Analysis of the surface of the murine SMIS, implementing embryonic fibroblasts (3T3) and epithelial cells (CMT-93) by scanning electron microscopy at two different magnifications. Higher magnification images identify membrane protrusions. Scale bar of the upper image indicates 2 µm while the lower displays 200 nm. **B** Immunostaining of superior and medial z-stack projections of murine spheroids consisting of a primary colonic myofibroblast (pMF) core (vimentin, green) and intestinal epithelial cells (CMT-93, CTNNB1, red). Nuclei are stained blue. **C** TEMs display the surface of the spheroids consisting of murine primary intestinal myofibroblasts (pMF) and epithelial cells (CMT-93) to identify membrane protrusions proving ordered microvilli formation. Scale bars of the upper image indicate 500 nm and of the lower image 200 nm. **D** Representative images of superior and medial z-stack projections of porcine SMIS. Localization of primary colonic fibroblasts is shown by immunostaining using vimentin (green) whereas epithelial cells by adherens junctions (IPEC-J2, CTNNB1, red). Scale bars indicate 100 µm. For green immunostaining DyLight 488 and for red staining DyLight 594 was used, whereas cell-nuclei were stained blue by DAPI in all IFs. Exposure time was identical for all spheroids and presented images are representative for three biological replicates tested

To evaluate reproducibility, we expanded the application of SMIS to mouse and pig species, demonstrating that a basic version of the human spheroid protocol can be universally applied across other species as well. Here, we assembled a fibroblast core and lined it solely with intestinal epithelial cells. For the murine model, we initially utilized NIH-3T3 embryonic fibroblasts to form the fibroblast core and employed the murine intestinal epithelial cell line CMT-93 to line the core. To characterize the spheroids, we stained them using IF with vimentin and CTNNB1 (Additional Fig. 3). In the nearly round spheroids, a prominent CTNNB1 signal was observed, indicating the fast formation of adherens junctions between CMT-93 cells that coated the entire surface of the murine SMIS. However, there was a weak vimentin signal detected in the core. For a more detailed examination, we further analyzed the spheroids using scanning and transmission electron microscopy (Fig. 5A). Similar to human electron micrographs, the murine spheroids with the NIH-3T3 core exhibited small, uneven microvilli-like membrane protrusions on the surface. However, in the murine spheroids, these finger-like protrusions appeared less irregular and displayed a more structured arrangement. Upon imaging at higher magnification, it became evident that these finger-like protrusions were composed of bundles of filaments, resembling the structure of microvilli. After successfully generating murine SMIS, we enhanced the model by substituting NIH-3T3 with primary intestinal myofibroblasts (pMF) isolated from murine colon tissue. Immunofluorescence staining revealed perfectly round-shaped spheroids. A strong vimentin signal in the pMF-core and CTNNB1 signal in the surrounding epithelial cells uncovered a monolayer of prismatic CMT-93 cells attached to the myofibroblasts (Fig. 5B). TEM images of the mouse SMIS with the pMF core showed regular membrane protrusions from the epithelial cells, identified as microvilli (Fig. 5C).

To extend the model for comparative studies related to zoonotic pathogens, porcine spheroids were generated using primary porcine colonic myofibroblasts [50] and the widely used porcine enterocyte cell line IPEC-J2. Characterization was performed using immunostaining of vimentin and CTNNB1. In the round-shaped spheroids, the adherens junctions revealed a complete enterocyte layer lining the myofibroblast core (Fig. 5D). However, only weak vimentin signal was detected, and it did not appear to be present evenly. Additionally, some epithelial cells seemed to be only partially connected to the core; vimentin artefacts due to spheroid fixation were visible.

## Discussion

In recent years, 3D cell culture has rapidly advanced and is now widely used in various biological research areas, showing promising outcomes. These models closely mimic animal and human tissue, aiding in reducing animal model usage. In our study, we developed advanced 3D cell cultures (SMIS) for human, mouse, and pig species. We characterized these spheroids using various microscopy techniques and compared them to traditional intestinal 2D cell cultures (Caco-2 cells). We also tested the spheroids' response to *C. jejuni* infection, comparing them to infected 2D cultures and in vivo data. This analysis aims to determine if 3D cultures could replace 2D models and serve as effective alternatives to animal testing.

Numerous methods exist for generating spheroids, including liquid overlay, hanging drops, microfluidics, or utilizing ultra-low attachment (ULA) plates [51–53]. Additionally, spheroids can be embedded in an extracellular matrix or cultured free-floating. Following an evaluation of various methods for forming the SMIS and considering factors such as cell seeding, spheroid formation, morphology, and reproducibility, we decided to utilize ULA plates with free-floating and modular self-assembling cellular aggregates in culture, which we were able to structure in a given order. This approach enabled the development of human SMIS, comprising a fibroblast core (NHDF neo) surrounded by a monolayer of enterocytes (Caco-2), goblet cells (HT-29/MTX-E12), and activated monocytes (A-THP-1), which completely lined the core. Nearly every cell on the outer layer made direct contact with the underlying fibroblasts. This achievement is noteworthy and has not been reported previously. In other studies involving multicellular spheroids, formation is achieved by simply mixing different cell types together without any directed organization [54, 55]. Further, in Kapałczyńska et al., for instance, the authors noted that, in their experience, cells of different phenotypes, such as fibroblasts and epithelial cells (as well as monocytes), did not integrate and form 3D structures together [1]. Moreover, the direct contact between enterocytes and other surrounding cell types facilitates not only epithelial-mesenchymal but also epithelial-immune cell interactions. Our intestinal model successfully integrates four important cell types to mimic the microarchitecture of the luminal intestinal surface as in the intestine, both epithelial cells and monocytes interact directly with mesenchymal cells of the Lamina propria. Simultaneously, relevant functional features such as microvilli formation are incorporated in SMIS. Caco-2 cells, in particular, exhibit a high degree of morphological and physiological similarity to the human intestinal epithelium. They are capable of forming a brush border, expressing typical metabolic enzymes, and efflux transporters [56, 57]. In 2D monolayers, these cells begin differentiation after seven days in culture and complete the process within 21 days [56, 58]. Notably, in our model, Caco-2 cells in contact with the mesenchymal core formed microvilli within just two days, as observed through scanning and transmission electron microscopy, rendering the spheroids a valuable model. Additionally, in contrast to our results, other studies have shown, that homotypic Caco-2 spheroids cultured for two to five days form round structures with a continuous smooth surface without microvilli [38]. This leads us to conclude that these mesenchymal-epithelial and mesenchymal-monocyte interactions are crucial factors driving the accelerated differentiation of enterocytes and the development of essential intestinal features, such as microvilli, within just two days of culture. In contrast, achieving full differentiation of Caco-2 cells for example requires up to 14 days when cultured in 2D [59].Apart from that, it's worth noting that intestinal organoids typically require 5–7 days to differentiate, emphasizing the time-efficiency of our model compared to both 2D and other 3D cell culture models [11]. We made the decision to culture the spheroids for only two days following the addition of enterocytes, goblet cells, and monocytes to minimize the occurrence of necrosis in the core caused by nutrient and oxygen deprivation. We observed slight structural instability in the spheroids after five days, likely due to necrotic processes within the spheroid [60, 61]. Unlike 2D monolayers, where cells receive uniform access to oxygen and nutrients, 3D spheroid models more accurately replicate the limited supply of these resources found in living tissues, particularly in the core. This constrained environment provides a more realistic simulation of physiological conditions, making the 3D model a more representative in vitro system [62].

The mammalian intestinal epithelium comprises various specialized types of proliferating and differentiated cells essential for maintaining intestinal functions. Proliferating cells located at the bottom of the crypts migrate upwards and undergo progressive differentiation into mature absorptive enterocytes. Genes such as *EGF* or *EPHB2* are predominantly expressed in undifferentiated, proliferative cells at the crypt base. EGF stimulates proliferation through growth factor signaling, while EPHB2, as a target of the Wnt-signaling pathway, regulates cell migration and proliferation upon activation by ligands [63–65]. Conversely, an opposing gradient in the expression of EPHB2 ligands, EFNB1, and EFNB2, can be observed. They exhibit high expression in differentiated cells of the upper parts of the crypt, particularly at the crypt-villus junction, which decreases towards the crypt base [64, 66]. We analyzed these four markers in SMIS and compared them with Caco-2 monolayers cultured for two days. *EFNB1* and *B2* exhibited significantly increased expression in the spheroids, suggesting a higher degree of differentiation in the intestinal cells of our spheroid model. However, *EGF* and *EPHB2* were also upregulated, indicating typical cell proliferative properties. This conclusion is further supported by the confirmed formation of microvilli, which are characteristic features of differentiated enterocytes. Thus, the enterocytes in the SMIS model achieve a higher degree of differentiation while simultaneously promoting proliferation, a combination that is typically challenging to attain within the same in vitro culture system. This underscores the advancement of the spheroids over 2D monolayers. The elevated proliferation of *EGF* and *EPHB2* found in SMIS may suggest that longer culture periods could be supported. However, as noted above, the structural instability after five days indicates that factors such as limited nutrient and oxygen diffusion might limit prolonged viability, despite active proliferation of the outer layer. A more detailed investigation into how SMIS simultaneously facilitates enhanced differentiation and increased proliferation will be addressed by our future studies.

To assess the absorptive capacities of the enterocytes in SMIS, we analyzed the expression of two transporters, the monosaccharide transporter *SGLT1* and the peptide transporter *PEPT1*. Both transporters are typically localized at the brush border of differentiated and absorptive enterocytes. [67, 68]. As expected, *SGLT1* expression was significantly upregulated in the spheroids compared to Caco-2 monolayers. However, *PEPT1* was significantly downregulated. This regulation might be attributed to increased EGF levels, as studies have shown that EGF can influence the expression of *PEPT1* [69, 70]. Although EGF treatment was not administered to Caco-2 cells in our study, considerable increase in *EGF* levels potentially provided by the fibroblast core could yield a similar effect. In the crypts, proliferation relies on various stimuli from the neighboring microenvironment, known as the niche. Studies have demonstrated that in the colon, intestinal mesenchymal cells establish an extra-epithelial niche by sustaining stimuli, including signals from EGF, Notch, and Wnt [71]. This may account for the increased expression of *EGF* and the subsequent reduction of *PEPT1* in SMIS. Nevertheless, we assert that the 3D co-culture model presented here exceeds conventional 2D models in sophistication, not solely in terms of the degree of differentiation. Taken together, the spheroids could serve as a novel and standardized research model not only for infection biology but also for other exposure studies. In addition to differentiation, the polarity of epithelial cells also plays a crucial role and directly impacts their physiology. The epithelial tissue of the mammalian intestinal mucosa is characterized by a simple, highly prismatic, columnar epithelium with a brush border containing microvilli on the apical side. We consistently observed a single layer of epithelial cells surrounding the spheroid core. Additionally, we observed cell–cell interactions between epithelial cells and the formation of microvilli exclusively at the apical side of the spheroids, highlighting the similarity to the luminal intestinal surface. However, given that SMIS are cultured for only two days, we were unable to detect columnar enterocytes as seen in vivo; instead, they exhibited a more isoprismatic shape. Investigating whether the morphology of enterocytes transitions to a more columnar shape with longer culture durations would be a stimulating point to explore in future studies. Moreover, the green-positive cells observed at the edge of the SMIS indicating vimentin expression, may suggest epithelial-to-mesenchymal transition (EMT). This could be attributed to a not fully differentiated status of the epithelial layer.

To assess SMIS as a suitable 3D infection model, we have implemented infection assays as a functional assay and infected the spheroids to investigate pathogen-epithelial interactions that were compared to our earlier in vivo studies [48, 49]. In intestinal infections, the initial tissue layers affected are the luminal layers of the mucosa, including the *Lamina epithelialis mucosae* and *Lamina propria mucosae*. Given that our model mimics these intestinal layers, the spheroids provide optimal conditions for an intestinal infection model. Moreover, the four cell types utilized have been demonstrated to be relevant for infection processes [72–75]. Our research and previous work [48, 49] has focused on the regulation of the molecular host cell response to infection, for which we initially developed a simpler 2D model. While conventional experiments on pathogen adhesion, invasion, and replication can still be effectively conducted using standard 2D cultures, the microarchitecture of SMIS, including the mesenchymal core, offers a unique opportunity to investigate cellular communication, transmigration and dissemination through subepithelial tissue. In this study, we focused on *C. jejuni* as an exemplary model pathogen. This choice is based on our prior studies involving this zoonotic pathogen, as well as the availability of in vivo infection samples for evaluation of SMIS and quality control [48, 49, 76]. Nonetheless, we are confident that the developed model will also be applicable to other bacterial pathogens.

We compared *C. jejuni*-infected SMIS to the naïve SMIS. In total, 10 immunological markers showed significant upregulation in the infected SMIS, with the highest levels observed in pro-inflammatory cytokines such as *IL1a*, *1b*, *6*, *8*, *TNFA* and *CCL2*. These cytokines play crucial roles in modulating cell-mediated immune responses following infection [77]. They orchestrate immune cell recruitment to the infection site, facilitating the control and elimination of intracellular pathogens such as *C. jejuni* through inflammation [78]. The upregulation of the investigated cytokines can thus be considered a representative defense mechanism of the SMIS against infection, a finding that has been investigated and published in numerous studies [79–82]. However, when comparing infected and uninfected Caco-2 monolayers, these findings may not be directly applicable. Among the six cytokines mentioned earlier, only *IL1a, IL12a* and *CCL2* show significant upregulation, albeit with a lower overall expression amplitude. The broader range of immunologically regulated factors with their intricate molecular mechanisms underscores the superiority of SMIS as an infection model over conventional 2D monolayers. Furthermore, all factors that are significantly upregulated in infected 2D model are also significantly increased in infected 3D models, but the reverse is not observed. The only exception is *IL12a* which is upregulated in the 2D but not in the spheroids. IL12a is predominantly expressed by macrophages and induces the cytolytic activity of immune cells. Yet, no studies have shown regulation of the cytokine in Caco-2 cells though an upregulation after *C. jejuni* infection was assessed before in human dendritic cells [82]. Increase of intracellular ST3GAL1 and B4GALT1 were detected in both infected 2D and 3D and supports our previously published data [49]. Only mRNA levels of *EGFR* are significantly downregulated in infected spheroids. The EGFR signaling pathway may play an important role in *C. jejuni* infection. Fibronectin-binding proteins from *C. jejuni* have been shown to activate the EGFR signaling pathway in host cells [83]. This activation leads to the Rho-GTPase CDC42-dependent formation of host cell membrane protrusions and is associated with increased invasion of *C. jejuni* [84]. Therefore, we hypothesize that the observed downregulation serves as a defense mechanism employed by the cells to impede pathogen invasion. We also compared non-infected human SMIS with Caco-2 monolayers and found upregulation of interleukins, TNFA, and ST3GAL1 in SMIS. This enhanced abundance of immunological markers suggests advantages from monocytic cell integration and intercellular interactions, underscoring the benefits of utilizing structured multicellular spheroids as a model system. We selected Caco-2 monolayers as a control for our SMIS model because they represent the traditional and widely used in vitro approach in intestinal infection biology. The added complexity of spheroids response to infection underscores the advantages of our model.

To evaluate how closely the molecular SMIS response mirrors in vivo infection and its potential to replace animal experiments, we compared our data with those from a murine model of human campylobacteriosis. This model was selected due to the limited availability of in vivo human samples, its extensive use in prior studies, and its suitability for comparing the immunopathological features of acute campylobacteriosis in humans with those observed in SMIS. For this analysis, we utilized cDNA from murine colon samples infected with *C. jejuni*, obtained from a previous study [49]. All immunological markers in the infected spheroids that were expressed in mice and had a significant upregulation more than two-fold (B4GALT1, CCL2, IL1a, IL1b, IL6, ST3GAL1, TNFA) were tested in *C. jejuni* infected colon samples and compared to naïve counterparts. All representative factors exhibited significant upregulation in the infected in vivo samples, indicating a close resemblance between the reported 3D infection model and the in vivo model. The gene expression patterns of numerous important immune factors mirror those observed in the animal experiment, highlighting the disparity with 2D monolayers. Additionally, we analyzed *EGFR*, the sole downregulated factor in the spheroids, which also showed downregulation in the colonic samples of infected mice. Based on these findings, we conclude that SMIS serve as an excellent model for studying intestinal infection biology, can be produced in a standardized and scalable manner, and are evidently superior to conventional monolayers. Furthermore, the comparable results from SMIS and the murine model of human campylobacteriosis suggest that SMIS not only represents gene expression patterns but also accurately reflects the functional aspects of the infection.

The core concept of our approach was to utilize readily accessible and expandable components, thus, we mainly used immortalized cell lines. Additionally, SMIS offer the advantage of growing in suspension, facilitating collection and fixation for downstream analyses such as IF, SEM or TEM. This offers a distinct advantage over other models, which e.g. involve feeder layers adhering to the culture flask, or two-compartment cultures that prevent direct physical contact between mesenchymal and epithelial cells. In our human model, we employed NHDF neo cells to establish the fibroblast core. However, previous research has pointed out genetic disparities between gastrointestinal and non-gastrointestinal fibroblasts. As NHDF neo cells are a human dermal fibroblast cell line, they might not precisely represent intestinal myofibroblasts [85]. Given the limited accessibility of human intestinal myofibroblasts, we optimized and adapted our model by incorporating murine and porcine primary myofibroblasts instead. Notably, the murine SMIS with a pMF core exhibited significantly enhanced differentiation of the epithelial layer with the same culture conditions as shown in the TEM images and immunofluorescence analyses. While the enterocytes in the human SMIS rapidly developed partly ordered microvilli, the murine SMIS with the pMF core exhibited more regular and ordered microvilli, making them more comparable to in vivo structures. Consequently, future studies should consider employing primary intestinal myofibroblasts also for human SMIS to enhance the model's physiological relevance. Moreover, we plan to incorporate monocytes into the spheroid core to better resemble intestinal microarchitecture. It's important to note that while the production of spheroids may be more labor-intensive compared to conventional 2D cell culture, they offer a unique level of complexity. Despite this, they are easy to reproduce, require less time, and are more cost-effective than organoids, other 3D models, or in vivo models, while still yielding comparable readouts.

As a versatile model, mammalian SMIS are not restricted solely to the study of bacterial infections; they hold potential for a wide range of scientific investigations. Particularly in the realm of nutrient absorption studies, this model offers practical utility due to its structural similarity to the luminal surface of mammalian intestinal organs, optimized cell differentiation, and the formation of microvilli. It is presumed that these characteristics contribute to improved absorptive properties of SMIS compared to traditional 2D cell cultures. Additionally, SMIS can prove beneficial in the examination of intestinal viral infections, pharmacological drug permeability and development, as well as toxicity testing. Certainly, the model holds the potential for expansion to mimic other organs by adjusting the cell types used to reflect the specific characteristics of the organ of interest. This adaptability opens doors for exploring various organ systems and their functions in a more physiologically relevant context. Indeed, our findings indicate the feasibility of extending the model to other species such as mice and pigs, thereby presenting numerous opportunities for its application. The reproducibility of the protocol for different species was successfully validated using different cell lines, providing consistent and reproducible results. Furthermore, our results also show the scalability of the protocol, which makes the use of the model suitable for medium-throughput.

Adhering to the principles of Reduction and Replacement outlined in the 3Rs framework, our mouse model can serve as a valuable tool for studying various pathological processes. Moreover, the mouse model offers the possibility of employing cell manipulation techniques to modify cellular responses, thereby enhancing its relevance and comparability for basic human and animal science. Pigs represent a promising model for investigations into nutrition and absorption, given the ecological and economic significance of these aspects in animal husbandry. Extensively studied for their physiological similarity to the human intestine and various analogous disease mechanisms [86], pig models offer valuable insights. However, differences in susceptibility to zoonotic pathogens present numerous molecular bases that we can explore using this model to translate preventive measures to humans. These attributes render them invaluable tools for advancing research and developing new therapeutic strategies.

In summary, we have successfully created structured multicellular intestinal spheroids for humans, mice, and pigs, that are fully standardized and scalable mimicking the luminal surface of the intestine in mammals. These spheroids exhibit typical intestinal traits observed in both differentiated and proliferating enterocytes. Among their diverse applications, SMIS represent an advanced model for investigating infection biology, yielding superior outcomes compared to conventional monolayers and demonstrating similarity to in vivo models.

## Conclusion

The aim of the study was to develop structured multicellular intestinal spheroids (SMIS) tailored for studying molecular basis of intestinal pathogenic infections. These innovative spheroids mimic the microarchitecture of the human intestinal mucosa by comprising four relevant cell types. For that, a fibroblast core is enveloped by an outer monolayer of enterocytes and goblet cells together with monocytic cells. These SMIS effectively emulate the in vivo architecture of the intestinal mucosal surface and manifest differentiated morphological characteristics within a mere two days of culture. By analyzing various aspects, we have shown that these spheroids attain heightened levels of differentiation compared to 2D monolayers. Moreover, SMIS serve as an optimized infection model to study intestinal pathogens like *Campylobacter jejuni*, surpassing the capabilities of traditional 2D cultures, and exhibit a regulatory pattern of immunological markers similar to in vivo infections. Remarkably, our protocol extends beyond human spheroids, demonstrating adaptability to other species such as mice and pigs.

## Materials and methods

### Cell lines and culture conditions

All cell lines were cultured in a humidified atmosphere at 37°C and 5% CO_2_. The human intestinal epithelial cells Caco-2 (DSMZ ACC 169) as well as the human colonic goblet cell model HT-29/MTX-E12 (ECACC 12040401), the murine intestinal cell line CMT-93 (ECACC 89111413), the murine primary colonic myofibroblasts (pMF), the murine embryonic fibroblast cell line NIH-3T3 (DSMZ ACC 59) and the porcine primary colonic myofibroblasts [50] were maintained in Dulbecco’s Modified Eagle’s Medium (DMEM) with 4.5 g/l Glucose and L-Glutamine (Gibco, Grand Island, NY, USA). The human dermal fibroblasts from the neonatal foreskin NHDF neo (Lonza, CC-2509) and porcine intestinal enterocytes IPEC-J2 (DSMZ ACC 701) were cultured in Gibco Dulbecco's Modified Eagle Medium: Nutrient Mixture F12 (DMEM: F12) with GlutaMAX supplement (Gibco) and an adherent subclone of the human monocyte-like cell line A-THP-1 [87] in RPMI Medium 1640 (Gibco) with 100 × HEPES (Carl Roth GmbH + Co. KG, Karlsruhe, Germany) and 10 × Sodium pyruvate (Sigma-Aldrich, Darmstadt, Germany). All media were supplemented with 10 µg/ml Gentamicin (Sigma-Aldrich) and 10% (v/v) fetal calf serum superior (Sigma-Aldrich). The cells were grown in 75 cm^2^ tissue culture flasks (Sarstedt, Nümbrecht, Germany) until approximately 80% confluence was reached. All cell lines were authenticated and recently tested for contaminations.

### Isolation of murine and porcine primary colonic myofibroblasts

The porcine intestinal myofibroblasts (clone #163) used in this study were isolated and described earlier [50]. Murine intestinal myofibroblasts (pMF) were isolated from wild-type NMRI mice by outgrowth culture using the porcine protocol [50] adjusted to the murine model. The murine colon tissue used for the isolation of pMF in this study was provided as excess material obtained from dissections conducted for teaching purposes at the OSZ Lise Meitner according to the European Guidelines for animal welfare (2010/63/ EU) following agreement by the commission for animal experiments headed by the “Landesamt für Gesundheit und Soziales” (LaGeSo, Berlin, registration number E0073/22). Colonic tissue was washed with warm medium (DMEM with 4.5 g/l Glucose and L-Glutamine, Gibco), supplemented with 10 µg/ml Gentamicin, 0.25 μg/ml Amphotericin B, 100 U/ml Penicillin and 100 μg/ml Streptomycin (Sigma-Aldrich). The lumen was cut open and the mucosa gently scraped off with a scalpel. The tissue was cut into small pieces (2 × 2 mm) and spread on a petri dish (Sarstedt). The dish was then filled with medium until the tissue was covered. The sections were then incubated at 37°C and 5% CO_2_. Medium was changed every 2–3 days. After approximately 4–5 days, first cells that morphologically looked like fibroblasts grew out of the tissue onto the plate. After the cells reached 80% confluence, they were transferred in a 75 cm^2^ tissue culture flasks (Sarstedt) and cultivated further.

### Characterization of murine primary colonic myofibroblasts

To identify whether the outgrown cells are in fact myofibroblasts, they were characterized by immunofluorescent staining of vimentin, a structural protein in mesenchymal cells, as well as alpha smooth muscle actin (α-SMA), a multifunctional protein of smooth muscle cells as described earlier [50]. Briefly, 5 × 10^4^ cells were seeded in each well of an 8-well cell culture chamber slide (Sarstedt) and incubated for two days. They were then fixed for 15 min at room temperature with Roti-Histofix (4% formaldehyde, Carl Roth). After blocking for 2 h at room temperature using 5% goat serum (v/v, Cell Signaling Technology (CST), Danvers, MA, USA) in phosphate-buffered saline (PBS, ROTIFair PBS 7.4, Carl Roth), the primary antibodies vimentin (Dako, Agilent Technologies, Waldbronn, Germany) and α-SMA (PROGEN Biotechnik GmbH, Heidelberg, Germany), were applied and incubated overnight. The next day, the cells were incubated with the matching secondary antibodies for 2 h at room temperature. Dilutions of all primary and secondary antibodies can be found in Additional Table 4 and 5. After three washes with PBS, the myofibroblasts were mounted on the glass microscope slide of the chamber slide in 50% glycerol in ddH_2_O. Immunostainings of the pMF can be found in the Additional Fig. 6.

### Assembly of SMIS

In order to generate ultra-low attachment surfaces for spheroid formation, suspension round-bottom 96-well cell culture plates (Sarstedt) were coated with Anti-Adherence Rinsing Solution (AARS, STEMCELL Technologies, Cologne, Germany, #07010) overnight at room temperature and washed with PBS the next day. For human SMIS, a core of fibroblasts was formed by using 5 × 10^3^ NHDF neo seeded on the coated wells and incubated for one day at 37°C and 5% CO_2_. The next day, 8 × 10^2^ Caco-2, 1 × 10^2^ HT-29/MTX-E12 and 1 × 10^2^ A-THP-1 were added to each well and observed under the microscope after two, four and six hours. When the cells were not arranged uniformly around the core, the cells were stirred up again using a pipette. The spheroids were then incubated for two days. For the murine intestinal spheroids 5 × 10^3^ pMF or 5 × 10^3^ NIH-3T3 were seeded on the AARS coated wells and incubated overnight. The next day, 1 × 10^3^ CMT-93 were added and multicellular spheroids formed after two days. For the porcine intestinal spheroids, 5 × 10^3^ porcine primary colonic myofibroblasts were firstly incubated for one day for the core and then 1 × 10^3^ IPEC-J2 were added and incubated for two days.

### Immunofluorescent staining of SMIS

Spheroids were fixed for 45 min at room temperature using Roti-Histofix (4% formaldehyde, Carl Roth). After three washes with PBS, antigen retrieval was carried out using a citrate buffer (10 mM sodium citrate dihydrate, 0.05% (v/v) TWEEN 20, pH 6) for 20 min at 97°C on a heating block. After switching off the heating block, the spheroids remained in the cooling block for a further 20 min. Subsequent steps were carried out at room temperature. The spheroids were then blocked for 3 h on a roller mixer using a blocking buffer (PBS with Triton-X-100 (1% v/v) and 5% goat serum (v/v) (CST)). They were then stained with the primary antibodies vimentin (Dako), CTNNB1 (Cell Signaling), CD68 (Novus Biologicals, bio-techne, Minneapolis, MN, USA) and PCNA (Abcam, Cambridge, UK) overnight. After the aspiration of the primary antibody solution and three washes with PBS, incubation of the secondary antibody solution (Thermo Fisher Scientific, Waltham, MA, USA, DyLight 488 goat-anti mouse; DyLight 594 goat anti rabbit) for three hours followed with three additional washes using PBS. Dilutions of all primary and secondary antibodies can be found in Additional Table 4 and 5. The nuclei were counterstained with 200 ng/ml 4′, 6-diamidin-2-phenylindol (DAPI, Sigma-Aldrich) diluted in PBS for 45 min. After three washes with PBS, the spheroids were mounted on a glass microscope slide in 50% glycerol in ddH_2_O. For microscopy, a Leica DMI6000B along with the Leica LAS-X-software (Leica, Wetzlar, Germany) was used. All images were taken under identical settings from at least 10 biological replicates. For the generation of 3D data, Z Stacks were used. Deconvolution was carried out by the LAS-X-integrated module.

### Scanning electron microscopy

Single human and murine spheroids were fixed in Eppendorf tubes using 2 ml of a 2.5% solution of glutaraldehyde (Sigma-Aldrich). Samples were washed two times in 0.1 M cacodylate buffer (Sigma-Aldrich). For dehydration, samples were passed through an increasing ethanol concentration gradient (30%, 50%, 70%, 90% and 99.8%). The obtained samples were sputtered with a carbon layer (Leica, EM ACE600) and observed with the Scanning Electron Microscope Auriga 60 (Carl Zeiss Microscopy, Oberkochen, Germany) as described earlier [88].

### Transmission electron microscopy

For thin section electron microscopy, the human and murine spheroids were individually fixed and stored in 2.5% glutaraldehyde. Prior to dehydration, samples were rinsed in 0.2 M sodium cacodylate (pH 7.2) and fixed in 2% aqueous osmium tetroxide for 2 h. After that, samples were washed in distilled water, dehydrated through a series of ethanol and impregnated, embedded and polymerized for 24 h at 60°C in Agar 100 epoxy resin. Ultra-thin (60–80 nm) sections were cut with diamond knives on a Leica ultramicrotome. For electron microscopy, sections were stained with 2% uranyl acetate and examined in a JEOL 1200EX transmission electron microscope operated at 80 kV as described earlier [88].

### Infection with *C. jejuni* 81–176

Human SMIS were generated as described above. 75 spheroids were then infected as previously described with *C. jejuni* strain 81–176 for six hours in a humidified atmosphere at 37°C and 5% CO_2_ using a multiplicity of infection (MOI) of 500 [49]. As controls, 75 non-infected spheroids only treated with PBS were considered. 15 spheroids from each group were randomly pooled to provide enough RNA for gene expression analysis and thus represented one biological replicate. Spheroids were washed in an Eppendorf tube five times using PBS, letting them sink to the bottom after every wash. To investigate differences between 3 and 2D cell culture, monolayers were infected as described earlier [49] with following adaptions: 3 × 10^5^ Caco-2 cells were seeded on 6 Well Plates (Sarstedt) and incubated for two days. The wells were then infected with *C. jejuni* strain 81–176 for six hours (also MOI of 500). As controls, non-infected monolayers were treated with PBS. After the infection time, wells were washed five times with PBS.

### RNA-isolation and RT-qPCR

For gene expression analysis, infected and non-infected spheroids and monolayers were lysed and RNA was isolated. For the spheroids, the *Quick*-RNA Micro Prep Kit (Zymo Research Europe GmbH, Freiburg, Germany) was used, for the Caco-2 cells the *Quick*-RNA Mini Prep Kit (Zymo Research Europe GmbH). 15 spheroids were pooled for one biological sample. RT-qPCR was performed as described earlier [48, 49]. As reference genes, *HPRT*, *B2M* and *PPIB* were selected and stability tested with geNorm [89]. For comparing SMIS with in vivo samples, murine cDNA generated from colon samples (LaGeSo, Berlin, registration number G0104/19) from our published study was used [49]. All oligonucleotides in this study were synthesized by Sigma-Aldrich. Sequences and primer concentrations can be found in Additional Table 7 and 8.

### Statistical analysis

Normal distribution of data was assessed by the Anderson–Darling test. The two-tailed unpaired t-test was used in this study to compare infected with non-infected control samples. All tests were conducted applying GraphPad Prism version 10.0.2 (GraphPad Software, La Jolla California USA, http://www.graphpad.com). Asterisks in figures summarize P values (*P ≤ 0.05; **P ≤ 0.01; ***P ≤ 0.001; ****P ≤ 0.0001).

## Supplementary Information

Supplementary Material 1

## Data Availability

No datasets were generated or analysed during the current study.
