# Supplementary material for: Structured multicellular intestinal spheroids (SMIS) as a standardized model for infection biology
Source: Gut Pathog. 2024 Sep 17;16:47. doi: 10.1186/s13099-024-00644-6 (PMC11406839; doi:10.1186/s13099-024-00644-6)
Supplement: Supplementary file 1 — Supplementary Material 1 [file 13099_2024_644_MOESM1_ESM.docx]

**Additional Files**

Structured multicellular intestinal spheroids (SMIS) as a standardized model for infection biology

Angelina Kraski ^1^, Paweł Migdał ^2^, Robert Klopfleisch ^3^, Clara Räckel ^1^, Jutta Sharbati ^4^, Markus M. Heimesaat ^5^, Thomas Alter ^6^, Carlos Hanisch ^7^, Greta Gölz ^6^, Ralf Einspanier ^1^, and Soroush Sharbati ^1*^

^1^ Freie Universität Berlin, Institute of Veterinary Biochemistry, Berlin, Germany

^2^ Wrocław University of Environmental and Life Sciences, Institute of Animal Husbandry and Breeding, Wrocław, Poland

^3^ Freie Universität Berlin, Institute of Veterinary Pathology, Berlin, Germany

^4^ OSZ Lise Meitner, School of Science, Berlin, Germany

^5^ Charité - Universitätsmedizin Berlin, corporate member of Freie Universität Berlin, Humboldt-Universität zu Berlin, and Berlin Institute of Health, Institute of Microbiology, Infectious Diseases and Immunology, Berlin, Germany

^6^ Freie Universität Berlin, Institute of Food Safety and Food Hygiene, Berlin, Germany

^7^ SGS Institut Fresenius, Berlin, Germany

* Correspondence: [soroush.sharbati@fu-berlin.de](mailto:Soroush.sharbati@fu-berlin.de); Tel.: +49 30 83862576

**Additional Files**

**
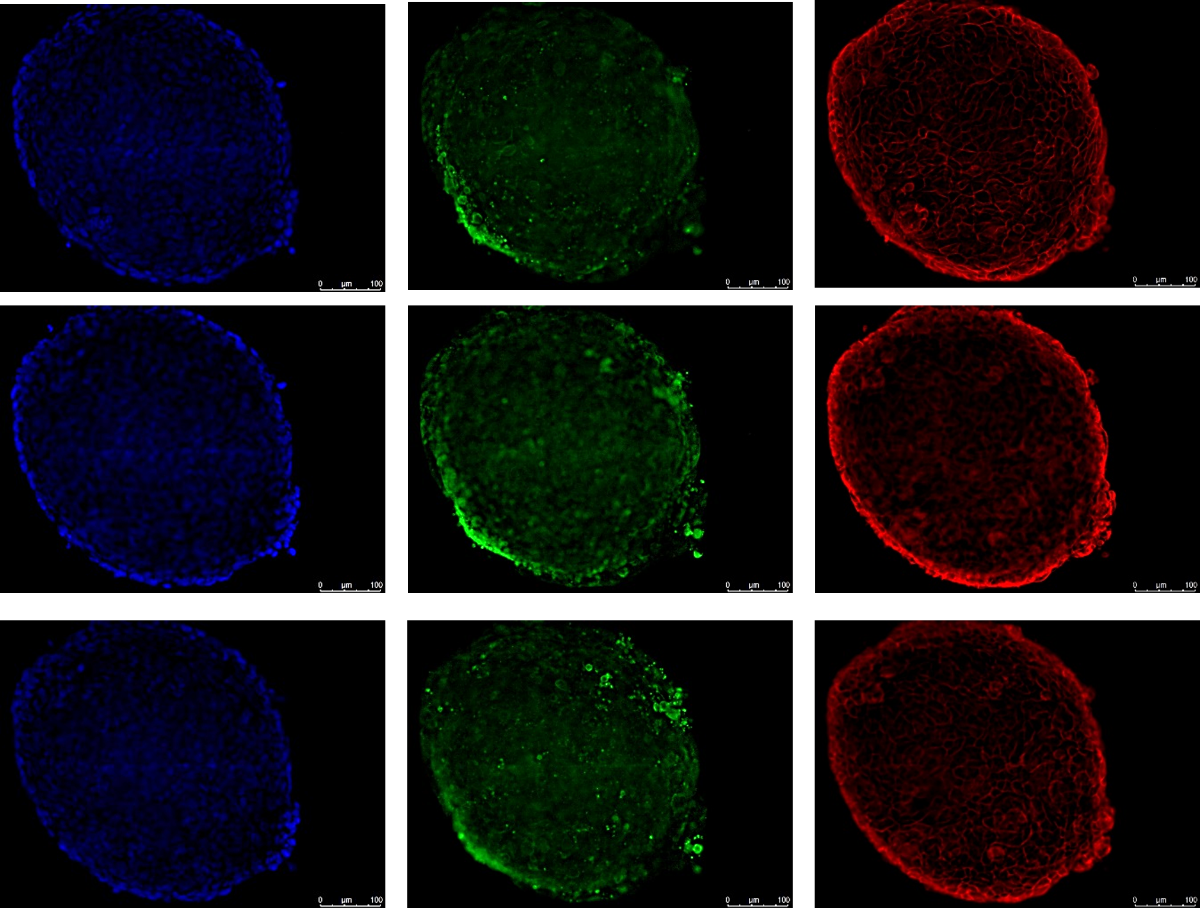
**

**Additional Figure 1**: Single staining images for each channel in Z-stack projections of human SMIS (pMF). Shown are Representative images of the localization of vimentin (green), adherens junctions between epithelial and goblet cells (CTNNB1, red) and cell-nuclei (blue). For green immunostaining DyLight 488 and for red staining DyLight 594 was used, whereas cell-nuclei were stained blue by DAPI Scale bars indicate 100 µm.


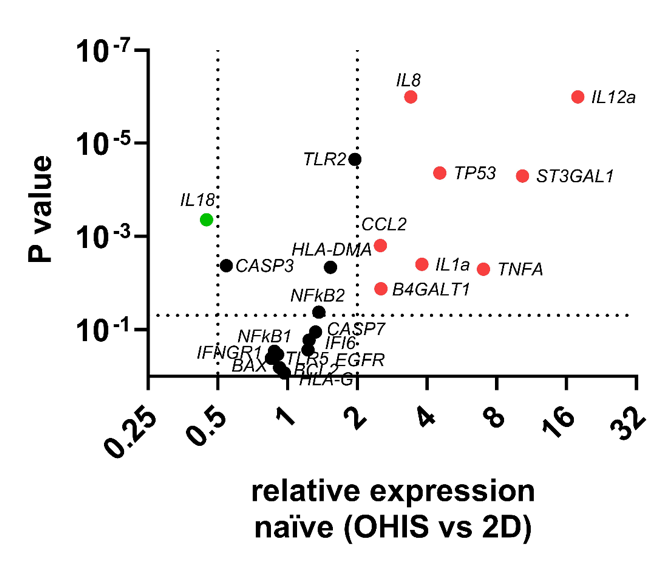

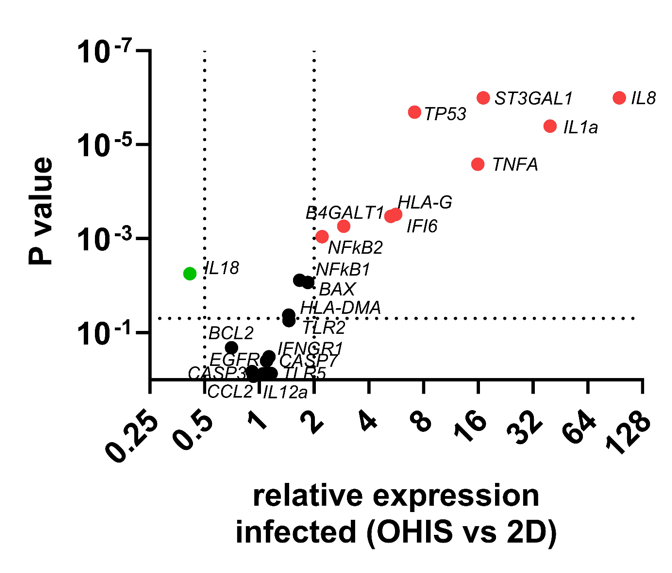


**Additional Figure 2**: Gene expression of immunological factors in human SMIS compared to Caco-2 monolayers. (A) Volcano Plot of differently expressed immunological genes in naïve SMIS compared to naïve Caco-2 cells. (B) Gene expression analysis of the immunological factors in *C. jejuni* infected SMIS, relatively calculated to *C. jejuni* infected Caco-2 monolayers and presented in a Volcano Plot.


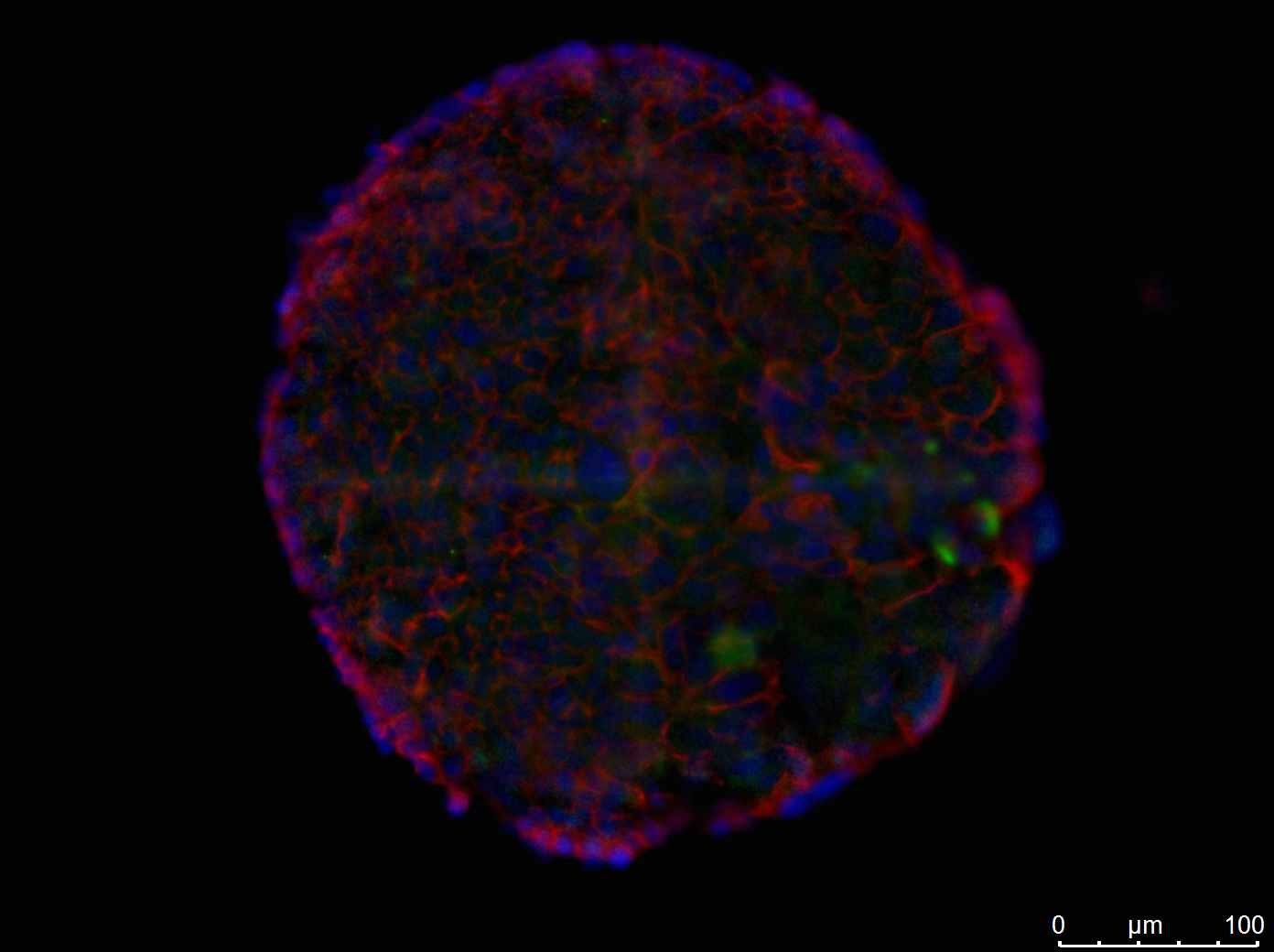

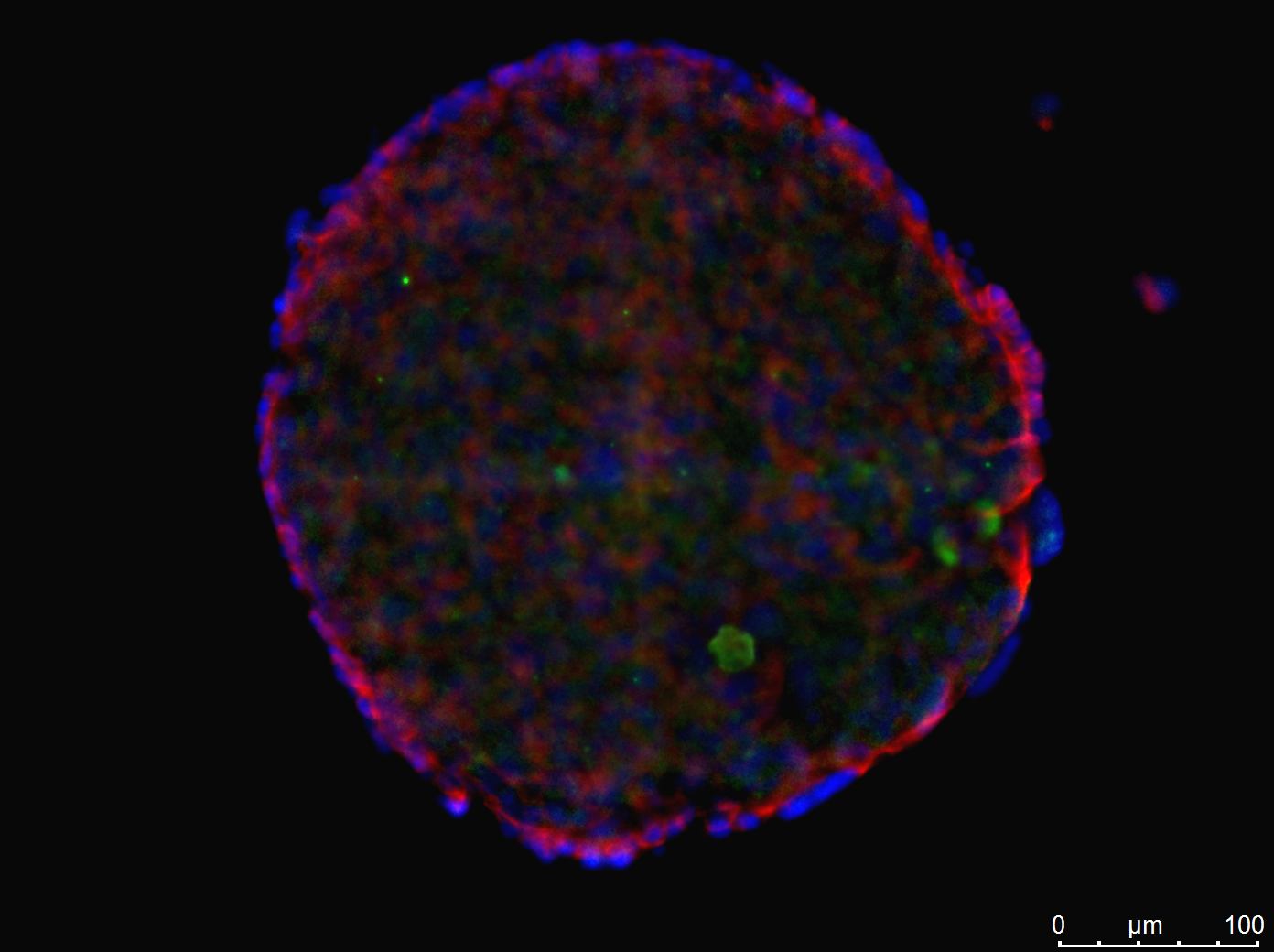


**Additional Figure 3**: Immunostaining of murine spheroids consisting of embryonic fibroblasts (3T3, vimentin, green) and intestinal epithelial cells (CMT-93, CTNNB1, red) in superior and medial z-stack projections. Cell nuclei are stained blue using DAPI.

**Additional Table 4**: Primary antibodies used in this study. Shown are name, company, order number and dilution of the antibodies.

| **Nr.** | **Name** | **Company** | **Order number** | **Dilution** |
| --- | --- | --- | --- | --- |
| 1 | Vimentin | Dako | M7020 | 1:100 |
| 2 | CTNNB1 | Cell Signaling | D10A8 | 1:100 |
| 3 | CD68 | Novus Biologicals | NBP2-37265 | 1:500 |
| 4 | PCNA | Abcam | ab92552 | 1:250 |
| 5 | α-SMA | PROGEN | 61001 | 1:400 |

**Additional Table 5**: Secondary antibodies used in this study. Shown are name, company, order number and dilution of the antibodies.

| **Nr.** | **Name** | **Company** | **Order number** | **Dilution** |
| --- | --- | --- | --- | --- |
| 1 | Goat anti-Rabbit IgG (H+L)Secondary Antibody  DyLight 488 | Thermo Fisher Scientific | 35552 | 1:800 |
| 2 | Goat anti-Rabbit IgG (H+L)Secondary Antibody  DyLight 594 | Thermo Fisher Scientific | 35560 | 1:400 |

**
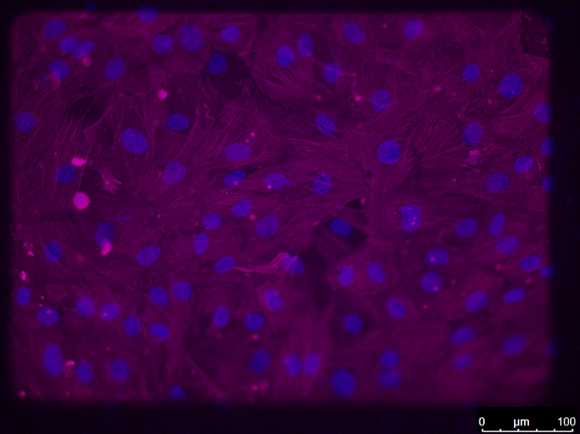
**
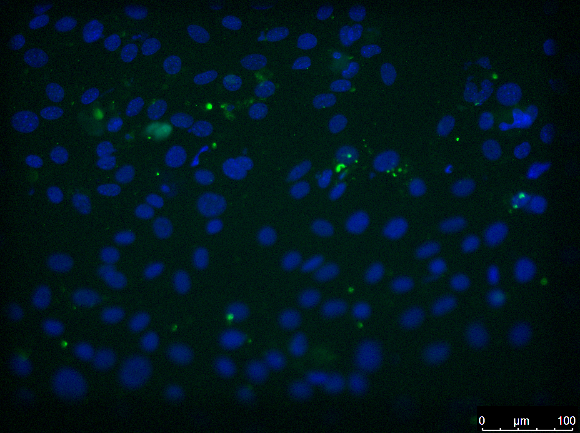


**Additional Figure 6**: Characterization of primary murine intestinal myofibroblasts (pMF). Positive immunofluorescent staining of alpha smooth muscle actin (α-SMA, magenta) and vimentin (green), counterstained with DAPI. These results confirm that the isolated cells are myofibroblasts. For green immunostaining DyLight 488 and for red staining DyLight 594 was used, whereas cell-nuclei were stained blue by DAPI Scale bars indicate 100 µm.

**Additional Table 7:** All oligonucleotides used in this study. Shown are the oligonucleotide sequences and primer concentrations of human mRNA primers.

| **Nr.** | **Gene** | **Oligo name** | **Sequence** | **Primer concentrations** |
| --- | --- | --- | --- | --- |
| 1 | B2M | hsa_B2M_fw | GTG CTC GCG CTA CTC TCT CT | 0.2 µM |
|  |  | hsa_B2M_rev | GGA TGG ATG AAA CCC AGA CA | 0.2 µM |
| 2 | B4GALT1 | hsa_B4GALT1_fw | ATG TTA TCA ACC AGG CGG GA | 0.2 µM |
|  |  | hsa_B4GALT1_rev | TGC CGT GGC TGT GAA AAA C | 0.2 µM |
| 3 | BAX | hsa_BAX_fw | CCA AGA AGC TGA GCG AGT GT | 0.2 µM |
|  |  | hsa_BAX_rev | CCA GTT GAA GTT GCC GTC AG | 0.2 µM |
| 4 | BCL2 | hsa_BCL2_fw | GCC CTG TGG ATG ACT GAG TA | 0.2 µM |
|  |  | hsa_BCL2_rev | TCA CTT GTG GCC CAG ATA GG | 0.2 µM |
| 5 | CASP3 | hsa_CASP3_fw | AGA ACT GGA CTG TGG CAT TGA G | 0.2 µM |
|  |  | hsa_CASP3_rev | GCT TGT CGG CAT ACT GTT TCA G | 0.2 µM |
| 6 | CASP7 | hsa_CASP7_fw | AGT GAC AGG TAT GGG CGT TCG | 0.2 µM |
|  |  | hsa_CASP7_rev | GCA TCT ATC CCC CCT AAA GTG G | 0.2 µM |
| 7 | CCL2 | hsa_CCL2_fw | GCC TCC AGC ATG AAA GTC TC | 0.2 µM |
|  |  | hsa_CCL2_rev | CAG ATC TCC TTG GCC ACA AT | 0.2 µM |
| 8 | EGF | hsa_EGF_fw | GAG ATG GGT GTC CCA GTG TG | 0.2 µM |
|  |  | hsa_EGF_rev | GGG TGG AGT AGA GTC AAG ACA G | 0.2 µM |
| 9 | EGFR | hsa_EGFR_fw | CCT GAG CTC TCT GAG TGC AAC | 0.2 µM |
|  |  | hsa_EGFR_rev | ACT GTT TCC AGA CAA GCC ACT | 0.2 µM |
| 10 | EPHB2 | hsa_EPHB2_fw | CAG ACC ATG ACA GAA GCC GA | 0.2 µM |
|  |  | hsa_EPHB2_rev | CAC ACG ATG GCG ATG ACA AC | 0.2 µM |
| 11 | EFNB1 | hsa_EFNB1_fw | AAG TTC CAG GAG TTC AGC CC | 0.2 µM |
|  |  | hsa_EFNB1_rev | ACA GCA TTG GGA TCT TGC CC | 0.2 µM |
| 12 | EFNB2 | hsa_EFNB2_fw | AAG ATC CAA CAA GAC GTC CAG A | 0.2 µM |
|  |  | hsa_EFNB2_rev | CCG TCT GTG CTA GAA CCT GG | 0.2 µM |
| 13 | HLA-DMA | hsa_HLA-DMA_fw | GCC CAA CAC TTT GGT CTG TT | 0.2 µM |
|  |  | hsa_HLA-DMA_rev | GTC CAT CGA CAG CTG AGA CA | 0.2 µM |
| 14 | HLA-G | hsa_HLA-G_fw | GCC TAC GAT GGC AAG GAT TA | 0.2 µM |
|  |  | hsa_HLA-G_rev | CCT CCA GGT AGG CTC TCC TT | 0.2 µM |
| 15 | HPRT | hsa_HPRT_fw | GAC CAG TCA ACA GGG GAC AT | 0.2 µM |
|  |  | hsa_HPRT_rev | CCT GAC CAA GGA AAG CAA AG | 0.2 µM |
| 16 | IFI6 | hsa_IFI6_fw | CGG GCT GAA GAT TGC TTC TC | 0.2 µM |
|  |  | hsa_IFI6_rev | TTT CTT ACC TGC CTC CAC CC | 0.2 µM |
| 17 | IL1a | hsa_IL1a_fw | ATC AGT ACC TCA CGG CTG CT | 0.2 µM |
|  |  | hsa_IL1a_rev | TGG GTA TCT CAG GCA TCT CC | 0.2 µM |
| 18 | IL1b | hsa_IL1b_fw | TCC AGG GAC AGG ATA TGG AG | 0.2 µM |
|  |  | hsa_IL1b_rev | TCT TTC AAC ACG CAG GAC AG | 0.2 µM |
| 19 | IL6 | hsa_IL6_fw | GAA AGC AGC AAA GAG GCA CT | 0.2 µM |
|  |  | hsa_IL6_rev | TTT TCA CCA GGC AAG TCT CC | 0.2 µM |
| 20 | IL8 | hsa_IL8_fw | GTG CAG TTT TGC CAA GGA GT | 0.2 µM |
|  |  | hsa_IL8_rev | CTC TGC ACC CAG TTT TCC TT | 0.2 µM |
| 21 | IL10 | hsa_IL10_fw | AAT AAG GTT TCT CAA GGG GCT | 0.2 µM |
|  |  | hsa_IL10_rev | AGA ACC AAG ACC CAG ACA TCA A | 0.2 µM |
| 22 | IL12a | hsa_IL12a_fw | TCA GCA ACA TGC TCC AGA AG | 0.2 µM |
|  |  | hsa_IL12a_rev | TAC TAA GGC ACA GGG CCA TC | 0.2 µM |
| 23 | IL18 | hsa_IL18_fw | CCT GGA ATC AGA TTA CTT TGG CA | 0.2 µM |
|  |  | hsa_IL18_rev | GTC CGG GGT GCA TTA TCT CT | 0.2 µM |
| 24 | IFNGR1 | hsa_IFNGR1_fw | CAT CAC GTC ATA CCA GCC ATT | 0.2 µM |
|  |  | hsa_IFNGR1_rev | TGT TCC ACT TTT CCT GGA TTG | 0.2 µM |
| 25 | NFKB1 | hsa_NFKB1_fw | CGG CTT CAG AAT GGC AGA AGA | 0.2 µM |
|  |  | hsa_NFKB1_rev | TTT GAA GGT ATG GGC CAT CTG T | 0.2 µM |
| 26 | NFKB2 | hsa_NFKB2_fw | ACG TAC CGA CAG ACA ACC TC | 0.2 µM |
|  |  | hsa_NFKB2_rev | GTC TTC CTT CAC CTC TGC TGT G | 0.2 µM |
| 27 | PEPT1 | hsa_PEPT1_fw | CCA AGT GCA TCG GTT TTG CC | 0.2 µM |
|  |  | hsa_PEPT1_rev | TGG TCA AAC AAG GCC CAG AA | 0.2 µM |
| 28 | PPIB | hsa_PPIB_fw | GGT GAT CTT TGG TCT CTT CGG | 0.2 µM |
|  |  | hsa_PPIB_rev | TAG ATG CTC TTT CCT CCT GTG | 0.2 µM |
| 29 | SGLT1 | hsa_SGLT1_fw | TGA GAC CCA CGA GCT CAT TC | 0.2 µM |
|  |  | hsa_SGLT1_rev | GGT GGA AAA CAT AGC CCA CAG | 0.2 µM |
| 30 | ST3GAL1 | hsa_ST3GAL1_fw | AAA CTC CAG CGT GTC TCC AG | 0.2 µM |
|  |  | hsa_ST3GAL1_rev | GTG ACC GTC CAT CTC TGG TC | 0.2 µM |
| 31 | TLR2 | hsa_TLR2_fw | AAG GGC AGC TCA GGA TCT TT | 0.2 µM |
|  |  | hsa_TLR2_rev | AGA CTG CCC AGG GAA GAA AA | 0.2 µM |
| 32 | TLR5 | hsa_TLR5_fw | CTG ACT CGT TCT CTG GGG TT | 0.2 µM |
|  |  | hsa_TLR5_rev | CCC GGA ACT TTG TGA CTG TG | 0.2 µM |
| 33 | TNFA | hsa_TNFA_fw | CCC TGA AAA CAA CCC TCA GA | 0.2 µM |
|  |  | hsa_TNFA_rev | AAG AGG CTG AGG AAC AAG CA | 0.2 µM |
| 34 | TP53 | hsa_TP53_fw | GTT CCG AGA GCT GAA TGA GG | 0.2 µM |
|  |  | hsa_TP53_rev | TCT GAG TCA GGC CCT TCT GT | 0.2 µM |

**Additional Table 8:** All oligonucleotides used in this study. Shown are the oligonucleotide sequences and primer concentrations of murine mRNA primers.

|  | **Gene** | **Oligo name** | **Sequence** | **Primer concentrations** |
| --- | --- | --- | --- | --- |
| 1 | B4GALT1 | mmu_B4GALT1_fw | AAGTTCGGGTTTAGCCTGCC | 0.2 µM |
|  |  | mmu_B4GALT1_rev | TTCTCCTCCCCAACCCCAAT | 0.2 µM |
| 2 | CCL2 | mmu_CCL2_fw | TGA CCC CAA GAA GGA ATG GG | 0.2 µM |
|  |  | mmu_CCL2_rev | ACC TTA GGG CAG ATG CAG TT | 0.2 µM |
| 3 | EGFR | mmu_EGFR_fw | GAG GTC CGC TAG AGA AAT GTC A | 0.2 µM |
|  |  | mmu_EGFR_rev | TGG GGC ATG TGC AGT GAT AG | 0.2 µM |
| 4 | HPRT | mmu_HPRT1_fw | AGTGTTGGATACAGGCCAGAC | 0.2 µM |
|  |  | mmu_HPRT1_rev | CGTGATTCAAATCCCTGAAGT | 0.2 µM |
| 5 | IL1a | mmu_IL1a_fw | GTC GGG AGG AGA CGA CTC TAA | 0.2 µM |
|  |  | mmu_IL1a_rev | GTT TCT GGC AAC TCC TTC AGC | 0.2 µM |
| 6 | IL1b | mmu_IL1b_fw | TGC CAC CTT TTG ACA GTG ATG | 0.2 µM |
|  |  | mmu_IL1b_rev | ATG TGC TGC TGC GAG ATT TG | 0.2 µM |
| 7 | IL6 | mmu_IL6_fw | GAC TGG GGA TGT CTG TAG CTC | 0.2 µM |
|  |  | mmu_IL6_rev | CAA CTG GAT GGA AGT CTC TTG C | 0.2 µM |
| 8 | SDHA | mmu_SDHA_fw | ACACAGACCTGGTGGAGACC | 0.2 µM |
|  |  | mmu_SDHA_rev | GCACAGTCAGCCTCATTCAA | 0.2 µM |
| 9 | ST3GAL1 | mmu_ST3GAL1_fw | GAGGATGAACAAGGCACCCA | 0.2 µM |
|  |  | mmu_ST3GAL1_rev | CACGGGGACATAGGTGTGAG | 0.2 µM |
| 10 | TNFɑ | mmu_TNFɑ_fw | ACC CTC ACA CTC ACA AAC CAC | 0.2 µM |
|  |  | mmu_TNFɑ_rev | CCC TTG AAG AGA ACC TGG GAG | 0.2 µM |
